# Long-term *in vivo* experimental investigation of porous carbonate apatite: light and transmission electron microscopy observations

**DOI:** 10.3389/fdmed.2026.1697338

**Published:** 2026-02-10

**Authors:** Keiichi Kanayama

**Affiliations:** Department of Dental Hygiene, University of Shizuoka Junior College, Shizuoka, Japan

**Keywords:** bioresorbable, carbonate apatite, fibroblast, osteoblast, osteoclast

## Abstract

**Introduction:**

As carbonate apatite (CA) has a composition similar to bone, it is considered a potential bone substitute. This study investigated *in vivo* resorption of porous CA disc using micro-CT and the qualitative features of multinucleated giant cells (MNGCs) and other cells on CA in bone defects using light microscopy and transmission electron microscopy (TEM).

**Methods:**

CA powder and commercial sugar with a grain size of 500–850 mm were mixed at an equal weight ratio. Compacted mixtures were placed in distilled water at 37°C for 40 min to allow the sugar to dissolve. Porous specimens were dried at room temperature overnight and then sintered at 700 °C for 1 h. Disc-shaped porous CA implants were inserted into femur bone defects in Wistar rats. Implanted CA was evaluated radiographically using micro-CT and histologically at 6 and 12 months, and ultrastructural observations were performed using TEM.

**Results:**

Bioresorption of porous CA increased over time, from 8.3% at 6 months to 13.9% at 12 months. At 12 months, new bone formation was also markedly increased inside porous CA. TEM analysis of the CA resorption process over the long term showed the involvement of mononuclear cells and MNGCs. These cells did not exhibit the typical ruffled border of osteoclasts.

**Conclusion:**

Resorption of porous CA continued for 1 year. New bone had formed up to the top of the porous CA by 12 months. In rat femur, MNGCs resorbed porous CA, and mononuclear cells, such as fibroblast-like cells, also contributed to CA resorption.

## 1 Introduction

Various bone substitutes have been created for use as scaffolds in clinical applications. The development of bone substitutes has garnered recent interest due to their advantages of consistent quality and safety (1). Bone substitutes used in dentistry are typically made of hydroxyapatite or *β*-tricalcium phosphate, each of which has specific characteristics. Hydroxyapatite is known for its stability, whereas *β*-tricalcium phosphate exhibits a higher resorption rate *in vivo* (2, 3).

The inorganic component of human bone is composed of carbonate apatite [CA; Ca_10−x_ (PO_4_)_6−x_ (OH)_2−x_ with 0 < x < 2]. The two carbonate substitution sites in the crystal lattice are the hydroxyl group (A-type) and the phosphate group (B-type). The fraction of B-type carbonate in bone mineral is more dominant than A-type. Synthetic CA should exhibit qualities comparable to those of autogenous bone (4). Carbonate substitution enhances biological affinity, protein adsorption, and resorption dynamics, making carbonate-containing apatite among the most biologically active calcium phosphate ceramics (5). Successful clinical trials have led to the approval of CA granules as an artificial bone substitute, and marketing of CA granules has begun in Japan and the US (6–8). In addition, CA granules tend to be resorbed after maxillary sinus floor elevation (9). When CA is applied clinically, it remains in the body for a certain period of time (10). The ability of bone tissue components and blood vessels to pass through the holes of porous bone substitutes results in high osteoconductivity and rapid bone regeneration (11–13). The porous surface of bone substitutes encourages a strong mechanical connection with the surrounding host bone (14, 15).

As CA has a porous structure and tends to promote bone growth, it has been successfully used as a bone substitute (16). CA provides more effective osteoclastic resorption than hydroxyapatite or β-tricalcium phosphate (17–19). Furthermore, porous CA enables sustained delivery of basic fibroblast growth factor, thereby enhancing bone augementation (20). CA is an effective bioresorbable bone substitute. A bone substitute should either promote bone healing through osteoconduction or be resorbed, degraded, and replaced by new bone. The replacement of CA with new bone emphasizes how osteoclasts coordinate the graft consolidation process (21). Mature osteoclasts are multinucleated giant cells (MNGCs) that originate from a hematopoietic progenitor and exhibit functional polarization. However, as osteoclast morphology varies during different stages of the cell cycle (22), how to characterize MNGCs on the surface of bone substitute materials is unclear. The presence or absence of a ruffled border, phagocytic activity, and analyses of cytoplasmic contents, as revealed by ultrastructural observations, could aid in characterizing these cells and enhance understanding of the biological effects of bone substitutes. However, the ultrastructural characteristics of cells involved in the degradation of CA and formation of new bone over the long term *in vivo* remain largely unexplored.

The aim of this study was to characterize the long-term cellular responses to sintered porous carbonate apatite implanted in rat bone defects. In addition to MNGCs, mononuclear cells, fibroblast-like cells, and osteoblast-lineage cells can provide an understanding of how different cell types contribute to the degradation of the material and the formation of new bone over extended implantation periods. We utilized both transmission electron microscopy (TEM) and conventional light microscopy for a comprehensive assessment. TEM is essential for visualizing ultrastructural features which are required to distinguish osteoclast-like resorption from macrophage-mediated phagocytic mechanisms. The present study investigated the ultrastructural basis of long-term resorption and remodeling of porous CA *in vivo*.

## 2 Materials and methods

### 2.1 Preparation of porous CA discs

CA was prepared basically as described previously and used to form porous CA disc implants (23). CA used in this study is a B-type carbonated apatite, in which carbonate ions substitute phosphate sites in the apatite lattice.

First, CA powder and commercial sugar (Chu-zara-to; DM Mitsui Sugar Co., Ltd., Tokyo, Japan) with a grain size ranging from 500 to 850 mm were combined in an equal weight ratio. To generate the porous material, compacted CA/sugar mixture was immersed in distilled water at 37 °C for 40 min to allow the sugar to dissolve. The CA/sugar mixture was compacted by cold isostatic pressing at 200 MPa prior to immersion in distilled water. After drying overnight, the porous material was sintered for 1 h at 700 °C.

### 2.2 Characterization of porous CA

**Figure 1:**
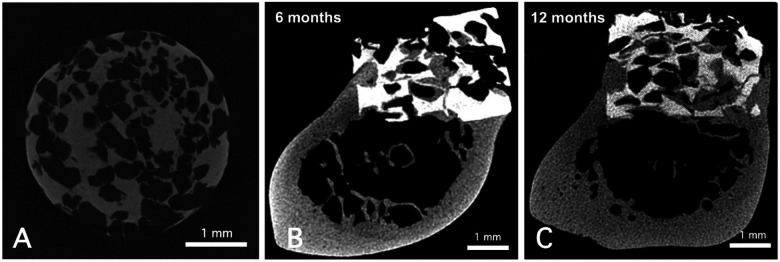
Representative micro-CT images of porous CA prior to implantation into the bone defect **(A)** scale bars: 1 mm. Representative micro-CT images of porous CA in rat femur defects at 6 **(B)** and 12 months **(C)** after surgical implantation. Scale bars indicate 1 mm.

The porous CA discs (diameter: 3.4 mm; height: 2.4 mm) were scanned using micro- focus x-ray computed tomography (micro-CT; SMX-90CT, Shimadzu, Japan). The porosity of the CA discs was approximately 52%, estimated from five reconstructed micro-CT images (Figure 1A). The micro-CT images were analyzed to determine pore size distribution, revealing an interconnected porous network with a mean pore diameter of 385 μm, ranging from 194 μm (minimum) to 635 μm (maximum). Porosity and pore size distribution were quantified using ImageJ software (version 1.54, NIH, Bethesda, MD, USA). The CA was manufactured by a sintering process, resulting in a highly crystalline structure (19). Previous studies have shown that such sintering reduces dissolution-driven resorption and tends to shift degradation toward phagocytic mechanisms mediated by multinucleated giant cells (18).

### 2.3 Surgical procedure

Six male Wistar rats (weight 300–350 g, 10 weeks old) were used as experimental animals. All rats were housed in a stable environment at 22 °C, 60% relative humidity, under a 12-h/12-h day/night cycle and with free access to water and food at Animal Facility of Asahi University. The study was approved by the Institutional Animal Care and Use Committee of Asahi University (Approval No.09-004, approved on July 14, 2009). Intraperitoneal Nembutal (pentobarbital sodium; Dainippon Abbot Laboratories, Tokyo, Japan) at a dose of 50 mg/kg anesthesia was administered prior to surgery. Following alcohol disinfection and shaving of the upper right limb of each animal, a sterile no. 15 surgical blade (Feather, Osaka, Japan) was utilized to make an incision in the skin. A sagittal defect through the distal femur was manually made using a 4.0-mm-diameter bone trephine bur (Dentech, Tokyo, Japan) drill in a sterile environment. Sterile porous CA discs were gently grasped with forceps and carefully inserted into the femoral bone defects using gentle manual pressure. Absorbable sutures were used to close the soft tissue in two layers, and the area around the wound was disinfected again. Ampicillin (2 mg/L), an oral antibiotic, was added to drinking water for 3 days after surgery. Without any restrictions, the animals were free to move. The implant period was 6 or 12 months. A total of 6 rats were used in this study, with 3 animals allocated to the 6-month time point and 3 animals to the 12-month time point. At the end of each implantation period, rats in that group were euthanized by administration of an overdose of pentobarbital.

### 2.4 Implant retrieval and micro-CT analysis

After euthanasia, the femur containing the implanted porous CA and surrounding tissues was harvested *en bloc* and fixed for 7 days in 4% paraformaldehyde in 0.1 M phosphate buffer. The right distal femurs were scanned using micro-CT before sectioning for light microscopy. Some porous CAs were also scanned using micro-CT before implantation. A slice thickness of approximately 10 mm was used for each micro-CT scan, and 120 images were collected at one time. After reconstructing the images in 1,024 × 1,024 pixel matrices, five or six randomly selected images were morphometrically analyzed using Mac SCOPE (Mitani Corp., Maruoka, Japan) to determine the material's degree of resorbability. The mean value per time point was used for statistical comparison. Resorption rate was calculated using the following equation:

Resorption%=(porearea+newbonearea/totalarea)×100–52.

Pre-implantation micro-CT scans of the porous CA discs were used to obtain the baseline pore area, reflecting the initial porosity of the material (approximately 52%). The resorption rate at 6 and 12 months was calculated as the relative increase in “pore area + new bone area” compared with this baseline value. Thus, the pre-implantation scans were essential for determining the degree of material resorption. Data are presented as mean ± standard deviation. The statistical significance of differences was determined using independent *t*-tests, and a significance level threshold of *p* < 0.05. The statistical analysis was performed using IBM SPSS Statistics version 24.0.

### 2.5 Preparation for light microscopy

Before embedding in paraffin, implanted porous CAs and surrounding tissues were decalcified for 14–20 days in 10% ethylenediamine tetraacetic acid (pH 7.4) and then dehydrated using a graded ethanol series. Paraffin sections (4 mm) were cut using a microtome and placed on silane-coated slides for enzyme histochemistry [tartrate-resistant acid phosphatase (TRAP)] and standard staining (hematoxylin and eosin). HE staining was performed on samples from the 6-month group (*n* = 1) and the 12-month group (*n* = 1). TRAP staining was applied to samples from the 12-month group (*n* = 1). Thus, not all samples underwent both staining procedures. TRAP was histochemically detected using the azo dye method. Tissue sections were incubated with 0.1% naphthol AS-BI phosphate and red violet LB salt. To verify tartrate resistance, the reaction was carried out in the presence of 50 mM sodium tartrate in 0.1M acetate buffer (pH 5.0).

### 2.6 Preparation for TEM

TEM analysis was performed exclusively on specimens retrieved 12 months after implantation. At 12 months after implantation, the rats were anesthetized with intravenous Nembutal (20 mg/kg body weight) and then perfused through the left ventricle and into the aorta with Ringer's solution followed by 2% glutaraldehyde/4% paraformaldehyde in 0.1 M cacodylate buffer containing 0.05% CaCl₂ (pH 7.4) for 15 min. The distal femur was removed, submerged in the same fixative for an additional 6 h, and then post-fixed for 2 h in 2% osmium tetroxide in cacodylate buffer (pH 7.4). After dehydration, the specimens were embedded in Taab 812 resin (Taab, London, UK). A diamond knife (DiATOME ultra 35°; Diatome, Biel, Switzerland) was used to cut ultra-thin sections (Dupont-Sorvall, Wilmington, DE, USA), which were then stained with uranyl acetate and lead citrate and examined under a JEM 1200EX electron microscope (JEOL, Tokyo, Japan).

## 3 Results

### 3.1 Micro-CT analysis

Representative micro-CT images of femur defects with porous CA discs are shown in Figures 1B,C. At 12 months after implantation, the resorption rate (13.9 ± 2.6%) was significantly higher (*p* < 0.05) than that at 6 months after implantation (8.3 ± 1.5%).

### 3.2 Histological analysis - light microscopy

**Figure 2:**
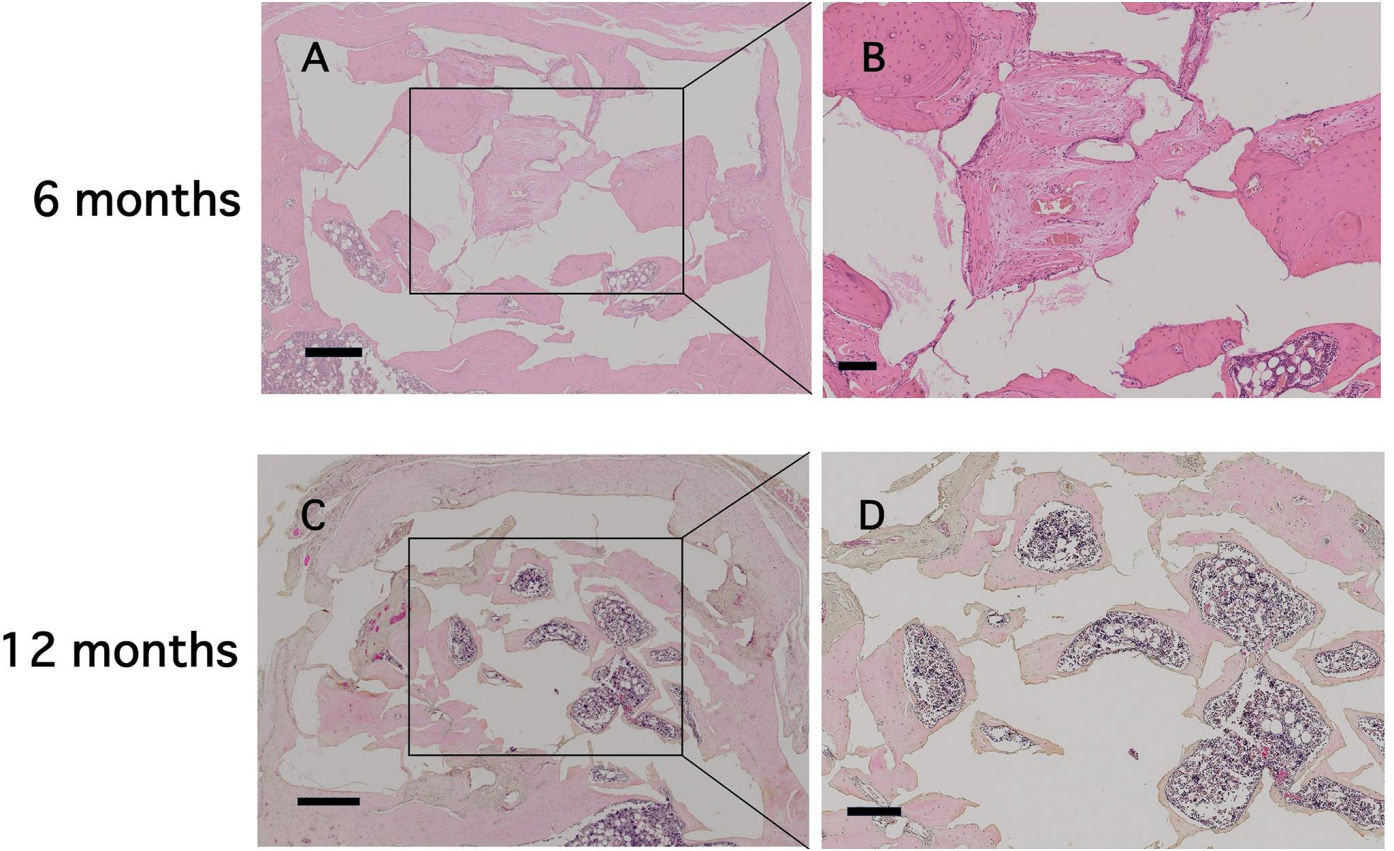
Light microscopic images (×2.5 to ×5) showing porous CA with regenerated bone tissue at 6 and 12 months. The specimens were stained with hematoxylin and eosin. Scale bars indicate **(A,C)** 500 μm and **(B,D)** 20 μm in low- and high-magnification images, respectively.

**Figure 3:**
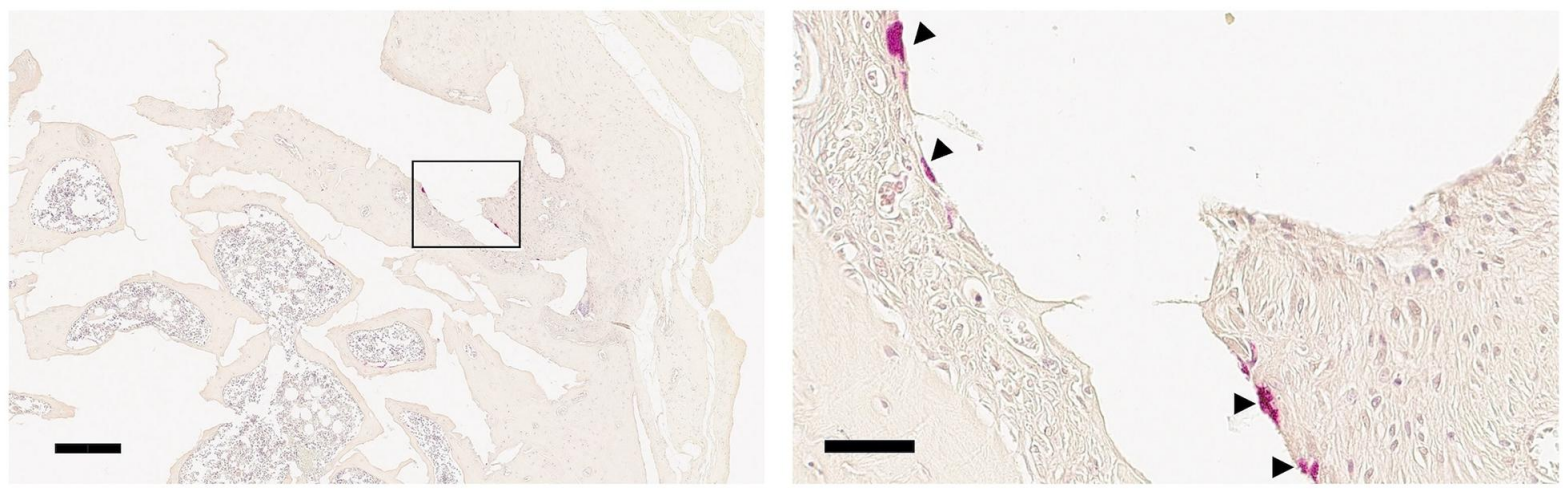
Light microscopic images (×5 to ×40) showing TRAP-positive cells (arrowheads) at 12 months post-implantation. The specimens were stained with TRAP. Scale bars indicate 100 μm and 50 μm in low- and high-magnification images, respectively.

In the present study, most of the porous CAs were filled with new bone in-growth, except for the center of the discs at 6 months (Figure 2A). The newly formed tissue in the central site consisted of fibrous connective tissue with blood vessels (Figure 2B). Bone marrow–like tissue was observed extending from the basal portion of the defect toward the central region (Figure 2A). At 12 months, the center of the discs was filled with newly formed bone (Figure 2C), and bone marrow–containing new bone extended to the upper portion of the defect by 12 months (Figure 2D). Approximately 80% of the pore area was filled with new bone (Figure 2C). A small number of TRAP-positive cells were observed at the surface of the porous CA discs. Both mononuclear and multinuclear TRAP-positive cells were observed (arrowheads in Figure 3). In this study, no signs of chronic or acute inflammation or infection were observed after 6 and 12 months of treatment.

### 3.3 TEM analysis

**Figure 4:**
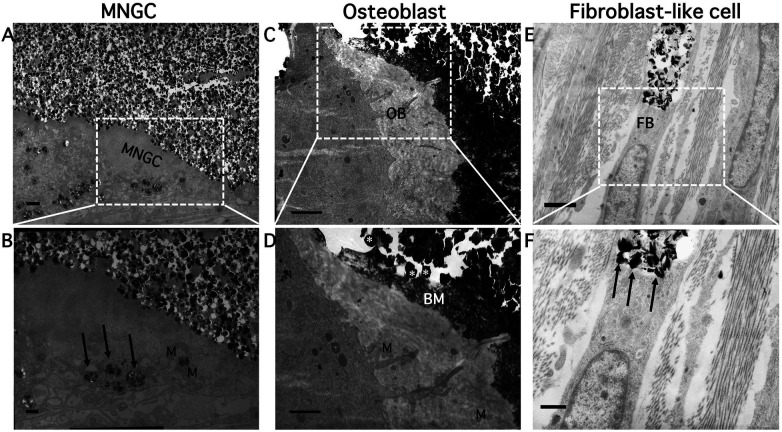
Observation of ultrastructural features using TEM. Lower panels show higher-magnification views of boxed regions in the upper panels. Scale bars: 1 μm in the upper panels and 500 nm in the lower panels. **(A)** CA particles were reabsorbed by MNGCs. **(B)** CA crystals in phagosomes (arrows) of MNGCs. Higher magnification showing mitochondria **(M)**. **(C)** Osteoblast (OB) adjacent to CA particles. As shown in **(D)**, the hexagonal-shaped CA crystals (asterisks) are clearly visible. Unmineralized new bone matrix (BM) with collagen fibers formed by an OB adjacent to CA. **(E)** Fibroblast (FB)-like cells also took up CA particles. **(F)** Some particles were observed in the vacuoles of these cells (arrows).

Mononuclear cells, such as fibroblast-like cells and MNGCs, which are rich in mitochondria and small vacuoles, were involved in the long-term resorption process *in vivo* (Figures 4A,E). Phagosomes containing fragmented CA crystals were identified within the cytoplasm of MNGCs (arrows in Figure 4B). Fibroblast-like mononuclear cells contained intracellular vesicles in which fragmented CA crystals were present, indicating phagocytic degradation (arrows in Figure 4F). MNGCs had small vacuoles and did not form a clear zone (a cytoplasmic region that indicates cell membrane adherence). Some irregular, rounded cytoplasmic protrusions facing the CA had penetrated into the gaps between the CA particles (Figures 4A,B). Endophagosomes were formed by surrounding CA particles via the development of pseudopodial cytoplasmic processes, unlike the typical ruffled border of bone-resorbing osteoclasts. The size and shape of CA particles post-phagocytosis were inconsistent. These findings suggested intracellular acidic degradation.

Crescent-shaped osteoblasts were also observed (Figure 4C). Osteoblasts with mitochondria secreted new bone matrix directly onto nearby CA particles, and these particles became embedded in the newly formed bone. The CA particles and new bone matrix were in close contact, without gaps (Figure 4D).

## 4 Discussion

Over a 12-month period, the present study quantitatively assessed the resorption rate of implanted porous CA discs and qualitatively evaluated cells observed on the surface of using both light microscopy and TEM. To the best of our knowledge, this is the first study reporting long-term (1-year) light microscopic and ultrastructural features of cells associated with implanted CA. A comprehensive understanding of these characteristics is crucial for forecasting the long-term outcomes of CA in clinical applications. The present study therefore aimed to quantify the long-term resorption of sintered porous CA and to characterize the ultrastructural and histological features of the cells interacting with the biomaterial.

The porous CA examined in the present study, which was prepared according to a previously reported method, exhibited a lower resorption rate than the material examined in the previous study (20). In this study, the resorption rate at 6 months was 8.3%, whereas the resorption rate at 3 months in the previous study was 9.7% (20). The height of the porous CA discs used in the present study was 20% greater, which could explain the difference in resorption rate. Nakagawa et al. found residual CA granules at sites of sinus floor augmentation 7 months after the procedure. The amount of residual CA granules was between 0% and 35% (7). Nagata et al. reported a mean CA granule resorption rate of 12.7% at 6 months after augmentation (9). The methods used to fabricate the porous CA primarily account for the difference between the present results and theirs. Their method was based on a dissolution-precipitation reaction from calcite blocks to low-crystalline CA (24), which is more rapidly resorbed *in vivo* than sintered CA. Low-crystalline CA described in Ishikawa et al (25), compares with sintered CA, which exhibit even broader XRD peaks, reflecting a lower degree of crystallinity. As no studies have compared fabrication methods involving sintering vs. dissolution-precipitation reaction, additional research is needed.

Early new bone formation reported in a previous study at the light microscopic level indicated that bone growth occurred around CA in contact with the defect edge at 2 weeks after the operation (20). Bone in-growth into the pores of CA was observed at 12 weeks, indicating partial new bone formation (20). In this study, the newly formed bone was mature and had bone marrow at both 6 and 12 months. By 12 months, new bone had formed up to the top of the porous CA discs, and TRAP-positive cells were observed. Kanazawa et al. reported complete resorption of CA blocks in osseous defects in rabbits within 1–1.5 years (26). The differences in results compared to our study can be attributed to differences in the fabrication methods, animal species, and implantation positions.

TEM analysis revealed MNGCs extended cytoplasmic protrusions resembling pseudopodia into the interstitial spaces between CA particles. The shape of MNGCs, however, differed somewhat from the characteristic ruffled-border appearance of bone-resorbing osteoclasts. These protrusions into CA adjacent to the MNGCs seemed to be simply cytoplasmic processes. The shape of the CA adjacent to or incorporated by MNGCs was generally unchanged or only partially degraded. This finding suggests that the extracellular dissolution of CA particles was not an active process and could have been the result of phagocytic resorption instead of being related to a specific osteoclast mechanism. Fibroblast-like cells have also been shown to play a role in the long-term resorption and degradation of CA particles (27). Previous ultrastructural studies have demonstrated that fibroblasts can internalize and phagocytose fine exogenous particles (27, 28). Cultured human oral fibroblasts have been shown to engulf dental amalgam particles, forming intracellular vacuoles coating electron-dense granules without degenerative change of cytoplasmic organelles (28). In the present study, the cells observed in Figure 4E exhibited spindle-shaped morphology and contained intracellular vacuoles enclosing degraded CA particles. These features are consistent with previously reported phagocytic behavior of fibroblast-like cells (27, 28). Reviews on calcium phosphate biomaterial degradation point out that for highly crystalline or low solubility CaP ceramics, cellular resorption often proceeds via particle dissolution followed by macrophage-mediated phagocytosis rather than osteoclast-mediated acidic resorption (29). Furthermore, recent evidence indicates that the composition of CaP materials strongly influences osteoclastogenesis: highly crystalline HA-rich materials impair osteoclast maturation, whereas more soluble β-TCP-rich formulations promote osteoclast differentiation and subsequent bone formation (30). These findings, together with our long-term *in vivo* data, suggest that the crystallinity and solubility of the sintered porous CA used in this study favor a macrophage-derived, phagocytic resorptive pathway. Under such conditions, MNGCs, or mononuclear cells with phagocytic capability, may constitute a biomaterial-induced resorptive phenotype, distinct from classical bone-resorbing osteoclasts. The morphology of metabolically active osteoblasts is either cuboidal or cylindrical. Osteoblasts facing the CA secreted new bone matrix components, including collagen fibrils. They did not exhibit the typical cuboidal shape of osteoblasts, but were instead crescent-shaped. Compared with active osteoblasts, the crescent-shaped cells appeared similar to lining cells, and intracellularly, they harbored a small number of mitochondria and rough endoplasmic reticulum (31). These characteristics showed that the observed “lining cells” were similar to quiescent osteoblasts in terms of morphology and distribution.

The translational relevance of these findings should be considered in the context of species differences between rodents and humans. Rats exhibit substantially higher bone turnover rates and more rapid remodeling activity than humans (32). Despite this, CA resorption remained slow and incomplete even after 12 months, suggesting that sintered CA may undergo similarly prolonged degradation in human bone. Clinical studies of CA granules in sinus floor augmentation have also reported persistent MNGC responses around residual particles (7–9). This indicates that macrophage-like MNGCs likely contribute to long-term CA degradation in humans. While absolute resorption rates cannot be directly extrapolated, the cellular mechanisms observed in the present rat model may provide a biologically plausible model for the long-term behavior of sintered CA in clinical applications.

This study has several limitations. A methodological limitation of this study is that TEM was not performed on regions pre-identified as TRAP-positive. As a result, it was not possible to directly correlate TRAP activity with ultrastructural features. Although TEM observations suggested phagocytic uptake of CA, quantitative measures of resorption activity—such as enzyme expression analysis, local pH monitoring—were not performed. Because this investigation was designed as a pilot *in vivo* study with the primary aim of characterizing the long-term ultrastructural features of cells associated with sintered CA, the sample size and methodology were not optimized for detailed biochemical quantification. In particular, the results should be interpreted with caution due to the limited number of animals.

## 5 Conclusion

CA resorption progressed gradually over a 12-month implantation period, accompanied by continuous cellular interactions at the material surface. MNGCs resorbed porous CA implanted in rat femur, with mononuclear cells, such as fibroblast-like cells, playing a role in CA resorption. Osteoblasts with a quiescent-like morphology contacted the surface of the CA remnant and secreted new bone matrix.

## Data Availability

The raw data supporting the conclusions of this article will be made available by the authors, without undue reservation.

## References

[1] Fukuba S Okada M Nohara K Iwata T. Alloplastic bone substitutes for periodontal and bone regeneration in dentistry: current status and prospects. Materials (Basel). (2021) 14:1096. 10.3390/ma14051096 33652888 PMC7956697

[2] Bouler JM Pilet P Gauthier O Verron E. Biphasic calcium phosphate ceramics for bone reconstruction: a review of biological response. Acta Biomater. (2017) 53:1–12. 10.1016/j.actbio.2017.01.076 28159720

[3] Jin P Liu L Cheng L Chen X Xi S Jiang T. Calcium-to-phosphorus releasing ratio affects osteoinductivity and osteoconductivity of calcium phosphate bioceramics in bone tissue engineering. Biomed Eng. (2023) 22:12. 10.1186/s12938-023-01067-1 PMC9912630 36759894

[4] Mano T Akita K Fukuda N Kamada K Kurio N Ishikawa K Histological comparison of three apatitic bone substitutes with different carbonate contents in alveolar bone defects in a beagle mandible with simultaneous implant installation. J Biomed Mater Res Part B Appl Biomater. (2020) 108:1450–9. 10.1002/jbm.b.34492 31622016

[5] LeGeros RZ. Properties of osteoconductive biomaterials: calcium phosphates. Clin Orthop Relat Res. (2002) 395:81–98. 10.1097/00003086-200202000-00009 11937868

[6] Kudoh K Fukuda N Kasugai S Tachikawa N Koyano K Matsushita Y Maxillary sinus floor augmentation using low-crystalline carbonate apatite granules with simultaneous implant installation: first-in-human clinical trial. J Oral Maxillofac Surg. (2019) 77:985. 10.1016/j.joms.2018.11.026 30597134

[7] Nakagawa T Kudoh K Fukuda N Kasugai S Tachikawa N Koyano K Application of low-crystalline carbonate apatite granules in 2-stage sinus floor augmentation: a prospective clinical trial and histomorphometric evaluation. J Periodontal Implant Sci. (2019) 49:382–96. 10.5051/jpis.2019.49.6.382 31886030 PMC6920036

[8] Ogino Y Ayukawa Y Tachikawa N Shimogishi M Miyamoto Y Kudoh K Staged sinus floor elevation using novel low- crystalline carbonate apatite granules: prospective results after 3-year functional loading. Mater (Basel). (2021) 14:5760. 10.3390/ma14195760 PMC8510057 34640156

[9] Nagata K Kamata M Okuhama Y Wakamori K Okubo M Tsuruoka H Volume change after maxillary sinus floor elevation with apatite carbonate and octacalcium phosphate. Int J Implant Dent. (2024) 10:7. 10.1186/s40729-023-00518-7 38329586 PMC10853090

[10] Imamura K Yoshida W Seshima F Aoki H Yamashita K Kitamura Y Periodontal regenerative therapy using recombinant human fibroblast growth factor (rhFGF)-2 in combination with carbonate apatite granules or rhFGF-2 alone: 12-month randomized controlled trial. Clin Oral Investig. (2024) 28:574. 10.1007/s00784-024-05979-7 39373727

[11] Ishikawa K Arifta TI Hayashi K Tsuru K. Fabrication and evaluation of interconnected porous carbonate apatite from alpha tricalcium phosphate spheres. J Biomed Mater Res B Appl Bio-mater. (2019) 107:269–77. 10.1002/jbm.b.34117 29577584

[12] Zhao YN Fan JJ Li ZQ Liu YW Wu YP Liu J. Effects of pore size on the osteoconductivity and mechanical properties of calcium phosphate cement in a rabbit model. Artif Organs. (2017) 41:199–204. 10.1111/aor.12742 27401022

[13] Kanayama K. Regeneration of class III furcation defects using porous carbonate apatite with basic fibroblast growth factor. J. Hard Tissue Biol. (2025) 34:23–8. 10.2485/jhtb.34.23

[14] Hannink G Arts JJC. Bioresorbability, porosity and mechanical strength of bone substitutes: what is optimal for bone regeneration. Injury. (2011) 42(Suppl 2):S22–5. 10.1016/j.injury.2011.06.008 21714966

[15] Karageorgiou V Kaplan D. Porosity of 3D biomaterial scaffolds and osteogenesis. Biomaterials. (2005) 26:5474–91. 10.1016/j.biomaterials.2005.02.002 15860204

[16] Hasegawa M Doi Y Uchida A. Cell-mediated bioresorption of sintered carbonate apatite in rabbits. J Bone Joint Surg Br. (2003) 85:142–7. 10.1302/0301-620x.85b1.13414 12585593

[17] Kanayama K Sriarj W Shimokawa H Ohya K Doi Y Shibutani T. Osteoclast and osteoblast activities on carbonate apatite plates in cell cultures. J Biomater Appl. (2011) 26:435–49. 10.1177/0885328210374672 20624844

[18] Doi Y Iwanaga H Shibutani T Moriwaki Y Iwayama Y. Osteoclastic responses to various calcium phosphates in cell cultures. J Biomed Mater Res. (1999) 47:424–33. 10.1002/(sici)1097-4636(19991205)47:3<424::aid-jbm19>3.0.co;2-0 10487896

[19] Doi Y Shibutani T Moriwaki Y Kajimoto T Iwayama Y. Sintered carbonate apatites as bioresorbable bone substitutes. J Biomed Mater Res. (1998) 39:603–10. 10.1002/(SICI)1097-4636(19980315)39:4<603::AID-JBM15>3.0.CO;2-7 9492222

[20] Kanayama K Kitago M Shiraki M Doi Y Shibutani T. Induction of new bone by bFGF-loaded porous carbonate apatite implants in femur defects in rats. Clin Oral Implants Res. (2009) 20:560–5. 10.1111/j.1600-0501.2008.01676.x 19515035

[21] Henriksen K Bollerslev J Everts V Karsdal MA. Osteoclast activity and subtypes as a function of physiology and pathology–implications for future treatments of osteoporosis. Endocr Rev. (2011) 32:31–63. 10.1210/er.2010-0006 20851921

[22] Everts V de Vries TJ Helfrich MH. Osteoclast heterogeneity: lessons from osteopetrosis and inflammatory conditions. Biochim Biophys Acta. (2009) 1792:757–65. 10.1016/j.bbadis.2009.05.004 19465118

[23] Doi Y Koda T Wakamatsu N Goto T Kamemizu H Moriwaki Y Influence of carbonate on sintering of apatites. J Dent Res. (1993) 72:1279–84. 10.1177/00220345930720090401 8360376

[24] Ishikawa K Hayashi K. Carbonate apatite artificial bone. Sci Technol Adv Mater. (2021) 22:683–94. 10.1080/14686996.2021.1947120 34434075 PMC8381965

[25] Ishikawa K Matsuya S Lin X Lei Z Yuasa T Miyamoto Y Fabrication of low crystalline B-type carbonate apatite block from low crystalline calcite block. JSC Jpn. (2010) 118:341–44. 10.2109/jcersj2.118.341

[26] Kanazawa M Tsuru K Fukuda N Sakemi Y Nakashima Y Ishikawa K. Evaluation of carbonate apatite blocks fabricated from dicalcium phosphate dihydrate blocks for reconstruction of rabbit femoral and tibial defects. J Mater Sci Mater Med. (2017) 28:85–95. 10.1007/s10856-017-5896-5 28456893

[27] Overgaard S Lind M Josephsen K Maunsbach AB Bünger C Søballe K. Resorption of hydroxyapatite and fluorapatite ceramic coatings on weight-bearing implants: a quantitative and morphological study in dogs. J Biomed Mater Res. (1998) 39:141–52. 10.1002/(sici)1097-4636(199801)39:1<141::aid-jbm16>3.0.co;2-i 9429105

[28] Onodera K Sugioka S Sasaki M Kumamoto H Ooya K. A fine structural study on the reaction of human fibroblasts to dental amalgam particles in a cell culture system. Tohoku Univ Dent J. (1995) 14:64–7. (In Japanese, English abstract)

[29] Sheikh Z Abdallah M-N Hanafi AA Misbahuddin S Rashid H Glogauer M. Mechanisms of *in vivo* degradation and resorption of calcium phosphate based biomaterials. Materials (Basel). (2015) 8:7913–25. 10.3390/ma8115430 28793687 PMC5458904

[30] Humbert P Kampleitner C De Lima J Brennan MÁ Lodoso-Torrecilla I Sadowska JM Phase composition of calcium phosphate materials affects bone formation by modulating osteoclastogenesis. Acta Biomater. (2024) 176:417–31. 10.1016/j.actbio.2024.01.022 38272200

[31] Hattori S. Structural features of ectopic bone-like tissue in porous hydroxyapatite blocks. Kokubyo Gakkai Zasshi. (2008) 75:120–37. (In Japanese, English abstract). 10.5357/koubyou.75.120 18634458

[32] Miller SC Bowman BM Jee WSS. Available animal models of osteopenia-small and large. Bone. (1995) 17(4 Suppl):343S–52. 10.1016/8756-3282(95)00284-k 8579907

